# Psychometric Properties of Preference-Based Measures for Economic Evaluation in Amyotrophic Lateral Sclerosis: A Systematic Review

**DOI:** 10.1155/2021/6681554

**Published:** 2021-01-28

**Authors:** Nicole Peters, Vanina Dal Bello-Haas, Tara Packham, Ava Mehdipour, Ayse Kuspinar

**Affiliations:** School of Rehabilitation Science, McMaster University, Hamilton, ON, Canada

## Abstract

**Objective:**

The aim of this review was to synthesize the psychometric properties of generic preference-based measures (PBMs) of health-related quality of life (HRQL) in Amyotrophic Lateral Sclerosis (ALS).

**Methods:**

A systematic review was conducted according to the Preferred Reporting Items for Systematic Reviews and Meta-Analyses guidelines. Four databases were searched from inception to April 2019: OVID Medline, Embase, PsycINFO, and CINAHL. Studies were included if (1) the sample represented individuals with ALS, (2) a generic PBM was utilized and reported on, and (3) information on the psychometric property of a generic PBM was provided.

**Results:**

Ninety-one articles were screened, and 39 full-text articles were reviewed. Seven full-text articles were included in this review. The mean age of participants ranged from 58.1 to 63.8 years, and mean time since diagnosis ranged from 20.5 to 44.6 months. Two generic PBMs were found, the EQ-5D-3L (*n* = 6) and the Quality of Well-Being Self-Administered (QWB-SA) scale (*n* = 1). Convergent validity of the EQ-5D-3L was large against a global scale of self-perceived health (*r* = 0.60) and small to large against ALS specific HRQL measures (*r* = 0.19 to 0.75). For the QWB-SA scale, correlations were small against a generic measure (*r* = 0.21) and large against ALS specific measures (*r* = 0.55). The EQ-5D-3L discriminated across different disease severity; however, floor effects were reported.

**Conclusion:**

This review highlights the need for more rigorously designed studies to assess the psychometric properties of generic PBMs in ALS and the development of an ALS specific PBM that adequately reflects the health concerns of individuals with ALS.

## 1. Introduction

Amyotrophic lateral sclerosis (ALS) is a neurodegenerative disease characterised by selective and progressive degeneration of voluntary motor neurons [1]. Adults with ALS have an overall mortality rate of 80% within the first 2 to 5 years after diagnosis and experience wide variability in disease severity and disease progression [2]. The disease affects more than 200,000 people worldwide in mid to late adulthood with an average age of onset of 55–66 years [3]. Signs and symptoms of ALS include (a) muscle weakness and atrophy resulting in loss of muscle control; (b) spasticity; (c) bulbar symptoms such as speech and swallowing difficulties; and (d) respiratory symptoms [4]. With disease progression and the resulting symptoms and loss of independence, the health-related quality of life (HRQL) of individuals with ALS is severely impacted [4–7].

HRQL instruments provide a structured way of including the patient's perspective when evaluating the influence of a disease and its treatments on one's physical, mental, and social well-being [5, 7, 8]. HRQL can be assessed using health profiles or preference-based measures (PBMs; also known as utility measures). Health profiles, such as the ALS Specific Quality of Life-Revised (ALSSQOL-R) scale, are scored by subscales and do not produce a single index score useful for economic evaluation purposes [5, 9, 10]. PBMs, on the other hand, are scored from 0.0 (death) to 1.0 (full health) and provide a single value of HRQL [9]. They can be used by researchers and policymakers for economic decision-making purposes to calculate quality-adjusted life years (QALYs) and determine the cost-effectiveness of interventions in ALS [9].

Existing PBMs used with individuals with ALS are generic and consist of measures such as the Short Form 6 Dimension (SF-6D) [11], Health Utilities Index Mark 3 (HUI3) [12], and EuroQol 5 Dimension (EQ-5D) (3 and 5 levels) [13, 14]. For some conditions, such as rheumatoid arthritis [15], cardiovascular disease [16], and various cancers [17], these measures have established estimates of reliability and validity. However, the reliability and validity of PBMs have not yet been summarized for ALS. As these measures were not developed specifically for individuals with ALS, it is important to assess their psychometric properties in this population [18]. This will assist in understanding whether the values obtained by the scoring system are valid and can be utilized by researchers and policy makers for clinical and cost-evaluation purposes. Therefore, the aim of this review was to synthesize the psychometric properties of generic PBMs of HRQL in ALS.

## 2. Methods

A structured search was conducted in accordance with Preferred Reporting Items for Systematic Reviews and Meta-Analyses (PRISMA) [19] reporting guidelines to identify possible articles that report information on the psychometric properties of PBMs of HRQL in ALS. COnsensus-based Standards for the selection of health Measurement INstruments (COSMIN) guidelines for systematic reviews of patient-reported outcome measures [20] were used to facilitate the understanding of a systematic review on PBMs and determine the quality of PBMs used.

### 2.1. Search Strategy

A research librarian (McMaster University, Hamilton, ON) was consulted for search strategy assistance a priori. Subsequently, a systematic search was conducted to identify all generic PBMs used in ALS. Four databases were searched: OVID Medline (1946 to April 9, 2019), Embase (1974 to April 9, 2019), PsycINFO (1806 to April 2019), and Cumulative Index to Nursing and Allied Health Literature (CINAHL, 1981 to April 9, 2019). Search terms were related to (i) Amyotrophic Lateral Sclerosis (ALS) AND (ii) a generic PBM: EuroQol Five Dimension (EQ-5D) (3 or 5 level), Health Utilities Index (HUI) (Mark 1, 2, or 3), SF-6D, the Assessment of Quality of Life (AQOL), 15-Dimension (15D) or Quality of Well-being (QWB) scale. Medical subject heading (MeSH) search terms and keywords were used for all databases and modified in accordance with the individual database search stipulations. See Supplementary File-Table 1 for the complete search strategy.

### 2.2. Study Selection

Two independent reviewers (NP and AM) identified potentially relevant articles by systematically screening titles/abstracts and then selecting full-text articles for inclusion. Reasons for exclusion were recorded, and if present, differences in responses between the two reviewers were discussed and a consensus reached. A third reviewer (AK) was consulted if a consensus was not reached. Studies were included if (1) the study sample represented individuals with ALS, (2) a generic PBM of HRQL was utilized and reported on, and (3) potentially relevant information on the psychometric property of a generic PBM was provided, whether this was their objective or not. Only full-text articles written in English or French and published in peer-reviewed journals were included in the review. Grey literature, conference proceedings, and abstracts were excluded.

### 2.3. Data Extraction

The following information was extracted independently, by two reviewers (NP and AM), from the full-text articles selected for data extraction: (i) study characteristics: author(s), year of publication, study design, study purpose, and study setting, (ii) sample characteristics: sample size (*N*), age, gender, time since diagnosis (months), ALS diagnosis, and disease severity, (iii) PBM(s) used (mean ± standard deviation (SD)), and (iv) psychometric properties. Specifically, the following metrics were sought from the included articles:

- Reliability(test-retest reliability): the extent to which scores of a measure have not changed over time, provided the characteristics being measured do not change [21, 22].
- Content validity: the degree to which the content of an instrument is an adequate reflection of the construct of interest [21].
- Construct validity
- Convergent validity: the degree to which scores of two measurement instruments relate when measuring a similar construct of interest [21, 23].
- Discriminative (known-groups) validity: the degree to which an instrument is able to discriminate between two groups that differ on the construct being measured [24].
- Predictive validity: the extent to which measurement instrument scores are an adequate reflection of a gold standard for the construct of interest in the future [18].
- Responsiveness: the ability of an instrument to detect change over time in the construct of interest [21].
- Floor/ceiling effect: the percentage of the sample obtaining scores at the lower and upper ends of the scale, respectively [18]; known as a form of interpretability that can affect the responsiveness of an instrument [18]. Floor and ceiling effects were deemed significant when percentage values >15% were seen [25].

### 2.4. Evaluation of Measurement Properties

The evaluation of measurement properties consisted of three steps. First, the methodological quality of studies was assessed using the relevant boxes for each measurement property included in the COSMIN Risk of Bias Checklist [26]. Second, the results of each study were rated against COSMIN's criteria for good measurement properties as either sufficient (+), insufficient (−), or indeterminate (?) [26]. Third, all results were rated and graded using COSMIN's modified Grading of Recommendations Assessment and Development and Evaluation (GRADE) approach (Supplementary File, Tables 2 & 3) [20, 26]. The evaluation of measurement properties could only be assessed for studies whose primary or secondary objective(s) was to evaluate the psychometric properties of a PBM [27].

The hypotheses derived were used to evaluate the psychometric properties when evaluating results against COSMIN's criteria for good measurement properties [26]. Reliability correlation coefficients were hypothesized to be greater or equal to 0.70 [18]. For measures assessing similar constructs (e.g., HRQL), we hypothesized large correlations of ≥0.50 [18, 28]. For measures assessing related, but dissimilar constructs (e.g. function/disease severity), we hypothesized a medium correlation of 0.30–0.49 [18, 28]. For discriminative (known-groups) validity, we hypothesized a significant difference in mean scores (*p* < 0.05) between groups of different predetermined variables (e.g., ALS severity levels) [26]. For predictive validity, areas under the curve (AUCs) were hypothesized to be greater than or equal to 0.70 [26]. Responsiveness was hypothesized to be significant at *p* < 0.05 or with an AUC ≥0.70 [26].

## 3. Results

### 3.1. Results of Search

**Figure 1:**
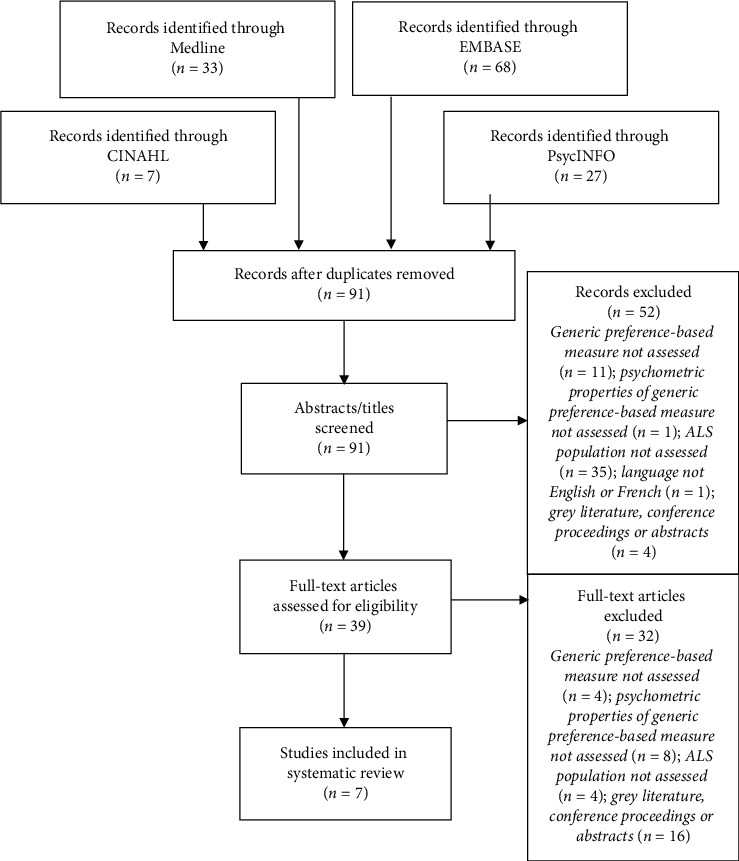
Flow diagram of article selection process (adapted from the PRISMA statement).^18^*ALS* Amyotrophic Lateral Sclerosis.

A total of 135 records were identified through the database searches. Forty-four records were removed due to duplication, resulting in a total of 91 articles for screening. Fifty-two articles were excluded during the initial screening of titles and abstracts. From this, 39 full-text articles were assessed for eligibility, whereby 32 of those articles were subsequently excluded. Articles were excluded if (i) a generic PBM was not assessed (*n* = 4), (ii) the psychometric properties of a generic PBM was not assessed (*n* = 8), (iii) the study did not report on or assess the population of interest (*n* = 4), and (iv) articles were grey literature, conference proceedings, or abstracts (*n* = 16). This left seven full-text articles for inclusion in the review.  Figure 1 outlines the complete review process.

### 3.2. Sample Characteristics

Table 1 presents key characteristics and psychometric properties from each study included in the review. Sample sizes across the seven studies ranged from 19 to 214 participants and 31% to 49% female. The mean participant age ranged from 58.1 to 63.8 years, and a mean time since ALS diagnosis of 20.5 to 44.6 months. ALS severity was classified according to (i) the ALS Functional Rating Scale-Revised (ALSFRS-R) (mean score = 32.63) [29]; (ii) the ALS Severity Scale (ALSSS) (mean score = 27.1) [31]; (iii) high or low severity classified as requiring caregiver assistance or not (75% of sample classified as high) [32]; or (iv) the ALS Health State Scale (ALS/HSS) (27–29% of sample classified as moderate or severe ALS) [33, 34]. If ALS severity was not reported, ALS diagnosis was classified using the El Escorial criteria with 21% to 47% of the sample classified as probable or definite ALS [30, 35].

### 3.3. Generic Preference-Based Measure(s) Used

Two PBMs were examined in the included studies: the EQ-5D-3L (*n* = 6) [29–34] and the QWB Self-Administered (QWB-SA) scale (*n* = 1) [35]. The EQ-5D-3L is a widely used generic PBM of HRQL [37]. It consists of five domains (mobility, self-care, usual activities, pain/discomfort, and anxiety/depression) and produces a single index score for health utility ranging from −0.594 for the worst possible health state to 1.0 for the best possible health state [38]. The QWB scale is an interview-administered scale that has been developed for self-administration (QWB-SA). It combines three scales of functioning with a measure of symptoms and problems and produces a single index score that ranges from 0.0 (death) to 1.0 (full function) [39].

Only one [35] of the seven included studies' primary purpose was to evaluate the psychometric property of a generic PBM, the QWB-SA scale. The remaining six [29–34] studies reported information on the psychometric properties of a generic PBM, the EQ-5D-3L; however, it was not the purpose of their study. Mean EQ-5D-3L scores ranged from 0.18 to 0.54, and a range of 37 to 214 individuals with ALS were included in these studies. A mean QWB-SA score of 0.43 was reported, and nineteen individuals with ALS were included in this study [35].

### 3.4. Psychometric Properties

Convergent validity, discriminative (known-groups) validity, and floor effects were reported in the seven included studies.

### 3.5. Convergent Validity

For the EQ-5D-3L, convergent validity was evaluated in four out of six studies (Table 2) [29–31, 33]. A large correlation of 0.60 with the EQVAS was reported in a single study (*n* = 77) [33]. Correlations with a disease-specific health profile, the ALS Assessment Questionnaire 40 (ALSAQ-40) subscales. ranged from small with the Eating and Drinking (ALSED) subscale (*r* = 0.19) to large with the Activities of Daily Living and Independence (ALSADL-I) subscale (*r* = −0.75) [33]. A large correlation of 0.72 with the disease-specific functional measure, ALSFRS-R, was reported in a smaller study (*n* = 46) [29]. A medium correlation of 0.43 was found with social support, as measured by the FSozU K-14 measure [29].

For the QWB-SA scale, one very small study (*n* = 19) evaluated convergent validity against a generic (SF-36) (*r* = 0.21) and disease-specific (Sickness Impact Profile ALS-19 (SIP/ALS-19)) (*r* = 0.55) health profile (Table 2) [35].

### 3.6. Discriminative (Known-Groups) Validity

For the EQ-5D-3L, all six studies evaluated known-groups validity (Table 1). This property was not assessed for the QWB-SA scale. The EQ-5D-3L was able to discriminate between patients across disease severity, with evidence of statistical differences in mean scores [29–34]. Of the three studies including mean values, the mean scores decreased (range = 0.65 to −0.01) with increasing disease severity [30, 32, 33]. Discriminative ability of the EQ-5D-3L was evident between people with bulbar or limb-onset ALS, with bulbar patients reporting a significantly higher EQ-5D-3L score (median = 46.4) than limb-onset patients (median = 14.9) [29]. Known-group validity was also established against two other neuromuscular diseases (i.e., myasthenia gravis (MG) and facioscapulohumeral muscular dystrophy (FSHD)), with lower scores reported in individuals with ALS [31] compared to individuals with MG and FSHD.

### 3.7. Floor Effects

Floor effects were reported for the EQ-5D-3L, where 54% to 92% of individuals with ALS reported moderate or severe problems across all five dimensions of the measure (Table 1) [29, 31, 34].

### 3.8. Evaluation of Psychometric Properties

Six out of seven studies could not be evaluated on the psychometric properties reported, as only one [35] of the seven studies' primary purpose was to evaluate the psychometric property of a generic PBM. For this study [35], a methodological quality analysis of the data resulted in a serious risk of bias, determined using COSMIN's risk of bias checklist [27]. In grading the quality of evidence using the GRADE approach and in accordance with hypotheses, there was serious inconsistency, very serious imprecision, and serious indirectness. This resulted in an overall rating of very low (Table 1).

## 4. Discussion

To our knowledge, this was the first study systematically reviewing the psychometric properties of generic PBMs in ALS. Across the seven studies included in this review, only the EQ-5D-3L and the QWB-SA scale were used in ALS. Furthermore, convergent validity, known-groups validity, and floor/ceiling effects were the only psychometric properties assessed for these measures in this population. Our review revealed that other important psychometric properties of PBMs (i.e., content validity, reliability, and responsiveness) have not yet been evaluated in ALS. Furthermore, none of the included studies, with one exception, were specifically designed to assess the psychometric properties of a generic PBM in the ALS population [35]. When the methodological quality of this study was assessed, the quality was graded as very low, preventing an accurate conclusion regarding the usability of the QWB-SA scale in the ALS population.

The EQ-5D-3L was highly correlated with the ALSFRS-R, an ALS specific functional rating scale reflective of disease severity; well exceeding our hypothesized correlation of less than 0.5 (for comparison of dissimilar constructs HRQL and disease severity). This is not entirely unexpected however as both the EQ-5D-3L [9] and the ALSFRS-R [40] contain similar domains, such as mobility and self-care, that are highly affected in ALS: this may explain the large correlations observed between the two measures [5]. Moreover, mobility is a domain that is greatly affected in various conditions, including ALS [41], due to its relation to independence and quality of life. As such, it is often included as a construct in many generic PBMs of HRQL.

The QWB-SA scale, however, may not be a generic measure that can be used in this population due to our study's findings and the unique nature of symptoms experienced by individuals with ALS. For example, the QWB-SA scale contains items that address mobility; however, the items are symptoms and limitations focused with little emphasis on ALS-relevant items such as functional mobility, speech, or pain [35, 42]. This could result in items that are not relevant to this population or even an underrepresentation of items that are relevant. Furthermore, the structure of the QWB-SA scale includes a style of item weighting that results in items relevant to individuals with ALS to contribute much less to the overall score. Furthermore, the QWB-SA scale was shown to weakly correlate with the generic SF-36 (*r* = 0.21) and strongly correlate with the disease-specific SIP/ALS-19 health profile (*r* = 0.55). Respectively, a correlation ≥0.50 and a correlation of 0.30–0.49 would be expected; however, the opposite was observed. Additionally, this was the only study included in the review with the primary purpose of psychometric evaluation. When the quality of evidence was assessed, it was deemed to be poor [27, 35]. As the QWB-SA scale was observed to correlate weakly with certain domains of the SF-36 that were similar in the EQ-5D-3 L and the ALSFRS-R, items included in the QWB-SA scale may not truly capture what is important to individuals with ALS or be the best tool for use in this population. However, as only one study has assessed this, further research is warranted in order to make accurate recommendations.

At a total score level, the EQ-5D-3L measure in ALS was able to discriminate between patients across disease severity as evidenced by significant differences in mean scores. However, at the individual item level, there is a prominent floor effect as majority of individuals reported moderate or severe problems in EQ-5D-3L domains, indicating the full scope of the disease is not being captured. This can affect the responsiveness of an instrument and the ability to accurately detect change over time [18]. For individuals with ALS, this is important to take note of as responsiveness is a critical property for assessing the cost-effectiveness of interventions in ALS [9]. Moreover, content validation, a fundamental component of validity, was not assessed in any of the studies. As such, generic PBMs may miss domains that are important or specific to individuals with ALS. For example, valued domains such as recreation and leisure activities and interpersonal relationships have been identified by individuals with ALS to be important to their quality of life [8]. However, these domains are not always assessed by generic PBMs. The development of an ALS-specific PBM would be one possible solution to help ensure that included domains reflect the health concerns of individuals with ALS.

PBMs, such as the EQ-5D-3L and the QWB-SA scale, were developed to provide evidence on the benefits or harms of a treatment on HRQL from the patient's perspective [9]. They provide a single index value of HRQL used to produce QALYs in order to evaluate the cost-effectiveness of interventions for a health condition [9]. PBMs can be of great use to patients, clinicians, and researchers alike; however, our results indicate there is limited evidence of their psychometric properties in ALS.

The EQ-5D consists of two parts. The first part (the descriptive system) assesses health in five domains: mobility, self-care, usual activities, pain/discomfort, and anxiety/depression. In addition to this, the EQ-5D contains a visual analogue scale (VAS) of self-rated health, scored from 0 to 100. The scores from the VAS cannot be used directly as weights in QALY calculations as they do not produce a single index value; however, the scores can be used as a subjective assessment of self-perceived health. It can provide clinicians and researchers with insight into how individuals perceive their overall health status, and how it changes over time with treatment. More recently, a 5-level version of the EQ-5D (EQ-5D-5L) was developed to improve the sensitivity of the measure and reduce ceiling effects [14]. The measure maintains the five domains from the EQ-5D-3L but expands from 3 to 5 response levels (no, some, moderate, severe, and extreme problems). The EQ-5D-5L defines a total of 3125 health states (5^5^)^14^, a substantial increase from the EQ-5D-3L with 243 health states (3^5^)^13^ and has been translated into more than 170 languages worldwide. For the EQ-5D-5L, each domain is scored from 1 to 5 and a utility value is derived from the five questions. To produce a single index score, a time-trade-off value set with general population preferences was recently developed for Canada [43]. The EQ-5D-5L may be useful in ALS; however, more studies should be conducted with the primary purpose of psychometric evaluation and utilization of this measure. This would result in a stronger conclusion regarding the appropriateness of the EQ-5D-5L for clinical research and economic evaluation.

One limitation for this systematic review is the small sample of studies included. As only one study's primary purpose was the psychometric evaluation of a generic PBM, there is limited evidence regarding the psychometric properties of generic PBMs in ALS. Another limitation is the use of only two generic PBMs in ALS; this may result in an imprecise representation and accuracy of generic PBMs' use in ALS.

## 5. Conclusion

To our knowledge, this is the first study systematically reviewing the psychometric properties of generic PBMs in ALS. The EQ-5D-3L was the most reported generic PBM. Although this measure demonstrated convergent and known-groups validity in ALS, significant floor effects were observed for all items, indicating that questions may not be appropriate for individuals with ALS. The only other measure used was the QWB-SA scale, which showed poor quality in its assessment of convergent validity and revealed items that are not relevant to individuals with ALS. Furthermore, there were psychometric properties of generic PBMs that have not been assessed in ALS, namely, content validity, reliability, and responsiveness. Therefore, our results highlight the need for more rigorously designed studies assessing the psychometric properties of generic PBMs in ALS or the development of an ALS specific PBM that reflects the health concerns of individuals with ALS.

## Figures and Tables

**Table 1:** Description of included studies.

Author (year)	Country	Study design	Study purpose	Study setting	Sample size (*N*)	Sample characteristics	ALS severity or diagnosis	Preference-based measurement used	Mean ± SD for measurement	Known-group validity	Convergent validity	Floor effect	Evaluation of measurement properties
Ilse et al. (2015) [29]	Germany	Cross-sectional study	To describe the relationship between HRQL using the EQ-5D, disease severity, and social support in patients with ALS	Outpatient clinic	*N* = 49	Age 63.8 ± 10.0, 49% female, disease duration 35.1 months ±36.3, time since diagnosis not presented, ALSFRS-R 32.6 ± 9.2 (range 0–48)	Severity classified according to ALSFRS-R 32.6 ± 9.2 (range 0–48)	EQ-5D-3L, EQVAS	EQ-5D*∗* 0.36 ± 0.29 EQVAS 42.8 ± 24.1	Bulbar-onset patients had a significantly higher EQ-5D score (median 46.4) than limb-onset patients (median 14.9) (*p*=0.034)	EQ-5D^a^:+FSozU K-14^b^ (r = 0.43, *p*=0.087), +BDI (r = −0.43), +ALSFRS-R (r = 0.72, *p* < 0.001)	61–86% of individuals with ALS reported moderate/severe problems in EQ-5D dimensions compared to 3-28% in general population	Not assessed: primary objective was not to assess psychometric properties of generic preference-based measure

Jones et al. (2014) [30]	UK	Longitudinal clinical trial	To assess whether ALS clinical staging could be used in cost-effectiveness analyses	10 outpatient clinics	*N* = 214	Age 58.1 ± 10.8, 31% female, time since diagnosis not presented, ALSFRS-R score not presented	ALS severity not presented, diagnosis classified according El Escorial criteria: definite (*n* = 82, 38%), probable (*n* = 80, 37%), probable laboratory supported ALS (n = 38, 17%), possible (n = 14, 5%)	EQ-5D-3L, EQVAS	EQ-5D score not presented for total sample	Mean EQ-5D scores decreased with increasing ALS severity^c^: from 0.65 (less severe) to 0.27 (more severe) (*p* < 0.001)	ALS clinical stage is a predictor of EQ5D score (*χ*² 145.08, *p* = 3.14 x 10^32^)	Not available	Not assessed: primary objective was not to assess psychometric properties of generic preference-based measure

Winter et al. (2010) [31]	Germany	Cross-sectional study	To compare HRQL in patients with ALS, FSHD, and MG and to identify the determinants of HRQL in each disease	7 outpatient clinics	Total *N* = 91, ALS *N* = 37	Age 59.6 ± 11.0, 43% female, time since diagnosis 39.7 months ± 73.7, ALSSS 27.1 ± 6.8 (range 0–40)	Severity classified according to the ALSSS 27.1 ± 6.8 (range 0–40)	EQ-5D-3L, EQVAS	EQ-5D*∗* 0.54 ± 0.32 (median 0.70), EQVAS 0.38 ± 0.15 (median 0.40)	Mean EQ-5D scores were significantly lower in ALS (0.54) compared to FSHD (0.75) and MG (0.89) (*p* < 0.01)	ALSSS significantly associated with EQ-5D (*p* < 0.01)	70–92% of individuals with ALS reported moderate/severe problems in EQ-5D dimensions compared to 3–28% in general population	Not assessed: primary objective was not to assess psychometric properties of generic preference-based measure

López-Bastida et al. (2009) [32]	Spain	Cross-sectional study	To determine the economic burden (direct and indirect costs) and assess HRQL in patients with ALS in Spain	Multiple outpatient clinics across 7 regions	*N* = 63	Age 59.1 ± 10.3, 48% female, time since diagnosis 44.6 months ±62.4, ALSFRS-R score not presented	Severity classified according to high^d^ or low^e^ severity: high severity (n = 47, 75%), low severity (n = 16, 25%)	EQ-5D-3L, EQVAS	EQ-5D*∗* 0.18 ± 0.22, EQVAS 0.29 ± 0.23	High severity: EQ-5D 0.12 ± 0.17, EQVAS 26 ± 22 low severity: EQ-5D 0.35 ± 0.27, EQVAS 38 ± 23 EQ-5D scores decreased with increasing ALS severity from 0.35 to 0.12 (*p* < 0.05)	Not available	Not available	Not assessed: primary objective was not to assess psychometric properties of generic preference-based measure

Green et al. (2003) [33]	UK	Cross-sectional study	To examine the relationship between disease severity, HRQL, and health state values in patients with MND	2 outpatient clinics	*N* = 77	Age 58.1 ± 12.1 (range 27–79), 36% female, time since diagnosis 25.3 months ±22.6 (range 1–112), ALSFRS-R score not presented	Severity classified according to the ALS/HSS: level 1 mild (n = 15, 20%), level 2 moderate (n = 21, 27%), level 3 severe (n = 22, 29%), level 4 terminal (n = 19, 25%)	EQ-5D-3L, EQVAS	EQ-5D*∗* 0.35 ± 0.35, (95% CI 0.27–0.43) (median 0.31), EQVAS 0.55 ± 0.22 (95% CI 0.5–0.6) (median 0.50)	Mean EQ-5D scores decreased with increasing ALS severity from 0.63 to −0.01 (*p* < 0.015)	EQ-5D^f^: +EQVAS (r = 0.60^g^); +ALSPM (r = −0.60^g^), +ALSADL/I (r = −0.75^g^), +ALSED (r = 0.19^g^), +ALSCOM (r = −0.32^g^), +ALSER (r = −0.43^g^)	Not available	Not assessed: primary objective was not to assess psychometric properties of generic preference-based measure

Kiebert et al. (2001) [34]	UK	Cross-sectional study	To assess HRQL and health state values in a sample of patients with different levels of severity of ALS	2 outpatient clinics	*N* = 77	Age 58.1 ± 12.1 (range 27–79), 36% female, time since diagnosis 25.3 months ±22.6 (range 1–112), ALSFRS-R score not presented	Severity classified according to the ALS/HSS: level 1 mild (n = 15, 20%), level 2 moderate (n = 21, 27%), level 3 severe (n = 22, 29%), level 4 terminal (n = 19, 25%)	EQ-5D-3L, EQVAS	EQ-5D score not presented for total sample, EQVAS 0.55 ± 0.22 (median 0.5)	The percentage of total sample who endorsed the worst response options of the EQ-5D increased with ALS severity across all dimensions; mean EQVAS scores decreased with increasing ALS severity from 0.74 to 0.37	Not available	54–80% of individuals with ALS reported moderate/severe problems in 4/5 EQ-5D dimensions (exception of 27% of people for *anxiety/depression*)	Not assessed: primary objective was not to assess psychometric properties of generic preference-based measure

Sherwood-Smith et al. (2000) [35]	USA	Cross-sectional study	To determine the concurrent validity of three self-administered HRQL questionnaires in patients with ALS	Outpatient clinic	*N* = 19	Age 60.5 (range 36–76), 42% female, time since diagnosis 20.5 months (range 2–62), ALSFRS-R score not presented, FVC 64% (range 17%–91%)	ALS severity not presented, diagnosis classified according to the El Escorial criteria: definite (n = 9, 47%), probable (n = 4, 21%), possible (n = 5, 26%), suspected (n = 1, 5%)	QWB-SA	QWB-SA 0.43 (range 0-1)	Not available	QWB^a^: +SIP/ALS (r = 0.55), +SF-36 (r = 0.21)	Not available	Methodological quality^h^: adequate Rating^i^: sufficient (inconsistent based on majority) grading of quality of evidence^j^: very low

HRQL: health-related quality of life, EQ-5D: EuroQol Five Dimension, EQVAS: EuroQol Visual Analogue Scale, ALSFRS-R: Amyotrophic Lateral Sclerosis Functional Rating Scale-Revised, BDI: Beck Depression Inventory, ALSSS: Amyotrophic Lateral Sclerosis Severity Scale, MG: myasthenia gravis, FSHD: facioscapulohumeral muscular dystrophy, MND: motor neuron disease; ALSAQ-40 :ALS Assessment Questionnaire 40 subscales; ALSPM: physical mobility, ALSADL/I: activities of daily living/independence; ALSED: eating and drinking; ALSCOM: communication; ALSER: emotional reactions, ALS/HSS: ALS Health State Scale; FVC: forced vital capacity, QWB-SA: Quality of Well-Being Self-Administered Scale, SF-36: Short Form 36, SIP/ALS-19: Sickness Impact Profile ALS-19. ^*∗*^Range of health utility scores from −0.306–0.885, with higher scores representing better health [36]. ^a^Spearman's rank correlation coefficient. ^b^Measures social support. ^c^Proposed clinical stages developed by Jones et al. estimated using ALSFRS-R scores and modified King's ALS staging system to indicate ALS severity. ^d^High severity: patients needed caregiver's assistance. ^e^Low severity: patients did not need caregiver's assistance. ^f^Pearson's product-moment correlation coefficient. ^g^Correlation is significant at the 0.01 level (two-tailed). ^h^Determined using COSMIN's risk of bias checklist^27^. ^i^Results rated against COSMIN's criteria for good measurement properties^26^: 50% of correlations (QWB: +SIP/ALS (*r* = 0.55), +SF-36 (*r* = 0.21)) in accordance with hypotheses, results rated as sufficient with an inconsistent rating from the majority of results. ^j^Determined using the GRADE approach. More details are described in detail in the COSMIN manual^26^.

**Table 2:** Convergent validity of the EQ-5D-3L and the QWB-SA scale.

	Comparison measure	Correlation (*r*)
EQ-5D-3L	EQVAS (global rating of self-perceived Health)^33^	0.60
	ALSAQ-40 (disease specific health Profile)^33^	
	PM	0.60
	ADL/I	0.75
	ED	0.19
	COM	0.32
	ER	0.43
	ALSFRS-R (disease specific functional Measure)^29^	0.72
	FSozU K-14 (social support)^29^	0.43
	BDI (depression)^29^	0.43
QWB-SA	SF-36 (generic health profile)^34^	0.21
	SIP/ALS-19 (disease specific health profile)^34^	0.55

EQ-5D-3L: EuroQol Five Dimension 3 Level, QWB-SA: Quality of Well-Being Self-Administered Scale, EQVAS: EuroQol Visual Analogue Scale, ALSAQ-40: ALS Assessment Questionnaire 40; subscales; PM: physical mobility; ADL/I: activities of daily living/independence; ED: eating and drinking; COM: communication; and ER: emotional reactions; ALSFRS-R: Amyotrophic Lateral Sclerosis Functional Rating Scale-Revised, FSozU K-14: Social Support; BDI: Beck Depression Inventory; SF-36: Short form 36; SIP/ALS-19: Sickness Impact Profile ALS-19.

## Data Availability

The data that support the findings of this study are available in this published article and in the supplementary material of this article.

## Supplementary Materials

Supplementary Materials Table 1: search strategy used for literature search. Table 2: COSMIN's modified GRADE approach for grading the quality of evidence. Table 3: instructions for downgrading risk of bias, inconsistency, and indirectness.
