# Supplementary material for: Psychometric Properties of Preference-Based Measures for Economic Evaluation in Amyotrophic Lateral Sclerosis: A Systematic Review
Source: Neurol Res Int. 2021 Jan 28;2021:6681554. doi: 10.1155/2021/6681554 (PMC7861917; doi:10.1155/2021/6681554)
Supplement: Supplementary Materials — Table 1: search strategy used for literature search. Table 2: COSMIN's modified GRADE approach for grading the quality of evidence. Table 3: instructions for downgrading risk of bias, inconsistency, and indirectness. [file 6681554.f1.docx]

Table 1. Search strategy used for literature search

| **Concept** | **Search Terms** |
| --- | --- |
| **Population** | Amyotrophic Lateral Sclerosis/  Amyotrophic lateral sclerosis.mp.  ALS.mp.  Lou Gehrig* disease*.mp.  Motor Neuron Disease*.mp. |
| **Preference-based measures**   - *Italics* = CINAHL only | EuroQol.mp.  EQ-5D*.mp.  *EQ 5D*.mp.*  EQ-5D-5L.mp.  EQ-5D-3L.mp.  EQ5D-5L.mp.  EQ5D-3L.mp.  EQ5D5L.mp.  EQ5D3L.mp.  *EQ 5D 5L*  *EQ 5D 3L*  *EQ5D 5L*  *EQ5D 3L*  Health utilit* index*.mp.  HUI.mp.  HUI1.mp.  HUI2.mp.  HUI3.mp.  HUI-1.mp.  HUI-2.mp.  HUI-3.mp.  HUI-I.mp.  HUI-II.mp.  HUI-III.mp.  *HUI 1.mp.*  *HUI 2.mp.*  *HUI 3.mp.*  *HUI I.mp.*  *HUI II.mp.*  *HUI III.mp.*  SF-6D*.mp.  SF6D*.mp.  Short Form 6D.mp.  Short Form Six Dimension.mp.  *SF 6D*.mp.*  *Short-Form 6D.mp.*  *Short-Form Six-Dimension.mp.*  AQOL.mp.  Assessment of quality of life.mp.  Generic utility* measure*.mp.  Generic preference based measure*.mp.  *Generic preference-based measure*.mp.*  Preference based measure*.mp.  *Preference-based measure*.mp.*  Quality of well-being.mp.  *Quality of well being.mp.*  QWB.mp.  15D*.mp.  15-D*.mp.  *15 D.mp.*  15-Dimension*.mp.  *15 Dimension*.mp.* |

.mp. – keyword designation for databases, * - open ended word for search (e.g. plural, hyphen etc.) to allow for as many possible results as possible

Table 2. COSMIN’s modified GRADE approach for grading the quality of evidence [1]

| **Quality of Evidence** | **Lower if** |
| --- | --- |
| High^a^ | Risk of bias  -1 Serious  -2 Very serious  -3 Extremely serious  Inconsistency  -1 Serious  -2 Very serious  Imprecision  -1 total n=50-100  -2 total n<50  Indirectness  -1 Serious  -2 Very serious |
| Moderate^b^ |  |
| Low^c^ |  |
| Very Low^d^ |  |

The starting point is the assumption that the quality of evidence is of high quality. The quality of evidence is subsequently downgraded to moderate, low or very low when there is a risk of bias (study quality), inconsistency (unexplained) in results, imprecision (from sample size) or indirect results. Information on the process is described in detail in the COSMIN user manual [1]. Definitions were adapted from the GRADE approach [2].

^a^Very confident that the true measurement property lies close to that of the estimate of the measurement property

^b^Moderately confident in the estimate of the measurement property; it is likely close to the true measurement property

^c^Confidence in the measurement property estimate is limited: it may be substantially different from the true measurement property

^d^Very little confidence in the measurement property estimate: it is likely to be substantially different from the true measurement property

n - sample size

Table 3. Instructions for downgrading risk of bias, inconsistency and indirectness [1]

| **Risk of bias** | **Downgrading for Risk of Bias** |
| --- | --- |
| No | There are multiple studies of at least adequate quality, or there is one study of very good quality available |
| Serious | There are multiple studies of doubtful quality available, or there is only one study of adequate quality |
| Very serious | There are multiple studies of inadequate quality, or there is only one study of doubtful quality available |
| Extremely serious | There is only one study of inadequate quality available |
| **Inconsistency*** | **Downgrading for Inconsistency** |
| Serious | If >50% of results were rated as sufficient according to COSMIN’s criteria for good measurement properties |
| Very serious | If <50% of results were rated as sufficient according to COSMIN’s criteria for good measurement properties |
| **Indirectness** | **Downgrading for Indirectness** |
| Serious | If other populations were also examined or none of the comparison measures examined quality of life or HRQL (for convergent validity and responsiveness) in the study. |
| Very serious | If other populations were also examined and none of the comparison measures examined quality of life or HRQL (for convergent validity and responsiveness) in the study. |

*only for inconsistent ratings

**References**

1. Mokkink, L. B., Prinsen, C. A. C., Patrick, D. L., Alonso, J., Bouter, L. M., De Vet, H. C. W., & Terwee, C. B. (2018). COSMIN methodology for systematic reviews of Patient ‐ Reported Outcome Measures ( PROMs ). User Manual, (February), 1–78.

2. Scünemann, H., Brożek, J., Guyatt, G., & Oxman, A. (Eds.). (2013). Handbook for grading the quality of evidence and the strength of recommendations using the GRADE approach. Retrieved from https://gdt.gradepro.org/app/handbook/handbook.html
